# RICNET: Retinex-Inspired Illumination Curve Estimation for Low-Light Enhancement in Industrial Welding Scenes

**DOI:** 10.3390/s25165192

**Published:** 2025-08-21

**Authors:** Chenbo Shi, Xiangyu Zhang, Delin Wang, Changsheng Zhu, Aiping Liu, Chun Zhang, Xiaobing Feng

**Affiliations:** 1 College of lntelligent Equipment, Shandong University of Science and Technology, Taian 271019, China; skd996523@sdust.edu.cn (C.S.); 202383230063@sdust.edu.cn (X.Z.); 202383230017@sdust.edu.cn (D.W.); zcs@sdust.edu.cn (C.Z.); 2 Beijing Botsing Technology Co., Ltd., Beijing 100176, China; lap@botsing.com

**Keywords:** real-time low-light enhancement, weld, zero-reference learning, computational photography, curve estimation

## Abstract

Feature tracking is essential for welding crawler robots’ trajectory planning. As welding often occurs in dark environments like pipelines or ship hulls, the system requires low-light image capture for laser tracking. However, such images typically have poor brightness and contrast, degrading both weld seam feature extraction and trajectory anomaly detection accuracy. To address this, we propose a Retinex-based low-light enhancement network tailored for cladding scenarios. The network features an illumination curve estimation module and requires no paired or unpaired reference images during training, alleviating the need for cladding-specific datasets. It adaptively adjusts brightness, restores image details, and effectively suppresses noise. Extensive experiments on public (LOLv1 and LOLv2) and self-collected weld datasets show that our method outperformed existing approaches in PSNR, SSIM, and LPIPS. Additionally, weld seam segmentation under low-light conditions achieved 95.1% IoU and 98.9% accuracy, confirming the method’s effectiveness for downstream tasks in robotic welding.

## 1. Introduction

Welding is a critical process in steel structure fabrication [1]. In recent years, driven by increasing welding quality standards and increasingly complex working environments, automated welding has gradually replaced traditional manual methods [2,3,4]. Welding robots demonstrate significant advantages in improving production efficiency and welding consistency; however, most existing systems still rely on a “teach-and-repeat” mode, which struggles to adapt to dynamic changes such as assembly errors and thermal deformation, resulting in reduced seam positioning and welding accuracy. Among these, crawling welding robots are particularly important in industrial applications due to their excellent mobility and environmental adaptability, with seam tracking tasks highly dependent on visual perception systems.

One of the core technical challenges in welding automation lies in the stable extraction of weld seam image features and real-time tracking capability under complex conditions. Although laser line corner tracking is the most commonly used weld seam perception method for crawling robots, intense arc light and complex ambient lighting severely interfere with visual systems, reducing tracking stability. To suppress such interference, optical filters or shading plates are often employed to block arc and background light. While this can enhance laser tracking robustness, it significantly reduces overall image brightness. This issue is especially pronounced in confined spaces such as tanks or ship hulls, where excessively dark images severely degrade the clarity and integrity of weld seam features, directly limiting subsequent seam recognition and processing. Even attempts to compensate by adding auxiliary light sources with wavelengths matching the laser fail to reconcile the contradiction between arc light suppression and sufficient image brightness, resulting in unstable weld feature extraction and ultimately impairing high-precision welding control.

**Figure 1:**
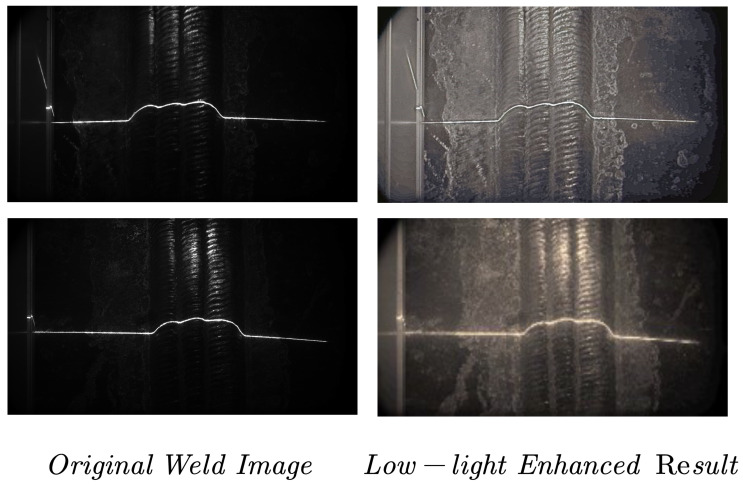
Comparison of low-light enhancement on welding seam images.

To address this critical issue, this paper introduces low-light image enhancement (LLIE) technology as an effective approach to improve the quality of weld seam feature information. As shown in Figure 1, LLIE can enhance image clarity and detail restoration while avoiding specular reflection artifacts, significantly improving weld seam detection, and tracking robustness under low-light conditions. Motivated by welding applications, we propose an LLIE-based weld image enhancement method that overcomes the limitations of traditional lighting schemes and improves the perceptual accuracy and operational stability of crawling welding robots in complex industrial environments.

Specifically, we designed an image enhancement framework based on Retinex theory, whose core idea is image decomposition—separating input images into invariant reflectance and variable illumination components. By constructing an adaptive zero-reference curve estimation mechanism for the illumination component, the framework dynamically compensates brightness to enhance overall luminance and contrast; meanwhile, it preserves structural details in the reflectance component to ensure the integrity of weld seam features. The network possesses clear physical interpretability and can be trained without relying on paired or unpaired industrial image datasets, significantly alleviating the problem of data scarcity in industrial scenarios and demonstrating strong practical value and generalization potential.

The experimental results demonstrate that our model achieves favorable subjective and objective performance on both publicly available datasets and our self-collected low-light weld seam dataset. Our main contributions are concluded as follows:

- An adaptive threshold-based low-light image enhancement framework based on Retinex theory is proposed, featuring a zero-reference illumination curve estimation mechanism. By dynamically perceiving local illumination characteristics, the framework adaptively adjusts the enhancement intensity. Without the need for paired training data, it effectively enhances low-light images while preserving fine details and suppressing noise, significantly improving the model’s robustness and generalization capability under complex lighting conditions.
- An innovative component-specific separation estimation strategy is proposed: Curve fitting is employed to estimate the illumination component, while image enhancement techniques are used to obtain the reflectance component. This unsupervised learning method can accurately model normal illumination and reflectance, producing more natural and realistic enhancement effects. The method not only significantly improves image brightness and contrast but also effectively preserves detail features, making it particularly suitable for real-world industrial scenarios where paired training data are unavailable.
- The proposed enhancement technology was successfully applied to the welding process of crawling robots. The experimental results demonstrate that this method significantly improves visual visibility and image contrast in low-light welding environments, fully showcasing its practical value in industrial welding applications.

The structure of the rest of this article is organized as follows: Section 2 reviews the related work on low-light enhancement. Section 3 describes the proposed method and architecture in detail. Section 4 presents extensive experiments and analyses to evaluate the performance of our approach. Finally, a discussion and general conclusion surrounding the algorithm are provided in Section 5.

## 2. Related Work

Over the decades, extensive low-light image enhancement (LLIE) methods have been presented, which can be roughly categorized into conventional methods and data-driven methods.

### 2.1. Traditional Methods

Techniques based on histogram equalization (HE) enhance image brightness by expanding the dynamic range. These methods include global histogram adjustment [5,6] and local histogram adjustment [7,8]. In addition to HE-based approaches, various methods grounded in Retinex theory [9] typically decompose an image into a reflectance component and an illumination component. Since the reflectance component is generally considered invariant under different lighting conditions, the problem of illumination enhancement is often transformed into an illumination estimation problem.

Building on Retinex theory, researchers have proposed multiple enhancement strategies. Wang et al. [10] introduced a method to preserve both naturalness and information content in images under non-uniform illumination. Fu et al. [11] employed a weighted variational model to jointly estimate the reflectance and illumination components, using the recovered reflectance component as the enhanced result. Guo et al. [12] generated an initial coarse illumination map by extracting the maximum intensity of pixel neighborhoods and further refined it using structural-aware priors. Li et al. [13] extended the Retinex model by incorporating noise factors, formulating illumination estimation as an optimization problem. Yuan and Sun [14] proposed an automatic exposure correction technique that globally optimizes an S-shaped curve for each image, mapping different regions to their optimal exposure ranges.

Unlike traditional methods that arbitrarily alter histogram distributions or rely on potentially inaccurate physical assumptions, the proposed approach adaptively enhances the illumination component through curve mapping based on Retinex theory while simultaneously improving the reflectance component. This method achieves natural visual effects without introducing distortion artifacts.

### 2.2. Data-Driven Methods

Data-driven low-light image enhancement methods can be broadly categorized into convolutional neural network (CNN)-based and generative adversarial network (GAN)-based approaches. Most CNN methods rely on paired supervised training data, which are often costly and labor-intensive to acquire. Such paired datasets are typically synthesized through automatic illumination degradation, adjustments to camera parameters during shooting, or post-processing. For example, LL-Net [15] and MBLLEN [16] use randomly gamma-corrected simulated data; the LOL dataset [17] is collected by varying exposure time and ISO settings; the MIT-Adobe FiveK dataset [18] contains 5000 raw images retouched by five professional editors; SID [19] provides paired low-light and normal-light RAW data; and [20] captures paired low-light RAW videos and their normal-light counterparts at corresponding frame rates.

Inspired by the Retinex model, many deep learning approaches use paired data supervision to estimate reflectance and illumination components for enhancement. Ren et al. [21] proposed a dual-stream deep hybrid network to jointly learn global content and salient structures. Wang et al. [22] trained an underexposed photo enhancement network on illumination maps retouched by experts. Zhang et al. [23] developed the KinD network to decompose images into illumination and reflectance components for brightness adjustment and degradation removal. However, Retinex-based deep methods are still limited by idealized assumptions, as the reflectance components of low-light and normal-light images differ significantly in real scenarios, affecting enhancement quality. To address this, our work specifically proposes denoising and enhancement for the reflectance component of low-light images to better restore image details.

Meanwhile, frequency domain analysis has gradually become a research focus. Xu et al. [24] proposed a frequency domain decomposition-based enhancement model that first restores low-frequency content and then enhances high-frequency details, trained on real noisy low-light images paired with standard sRGB images. Recently, a new trend in low-light enhancement is the integration of Transformer architectures, frequency domain representations, and diffusion models. LLHFN [25] incorporates homomorphic filtering into a deep learning framework to separate illumination and reflectance in the frequency domain, effectively improving structural fidelity in low-light images. MCATD [26] combines multi-scale contextual attention Transformers with diffusion models to capture long-range dependencies and progressively refine image details, achieving robust generalization under varying illumination conditions; FFTFormer [27] designs a spatial-frequency hybrid CNN–Transformer architecture to suppress noise and enhance details. These studies have advanced the application of frequency domain analysis and Transformer techniques in low-light image enhancement.

Nevertheless, the high cost of acquiring paired data and the potential artificial artifacts limit the generalizability of CNN-based methods in diverse real-world lighting conditions, often resulting in artifacts and color shifts. To tackle this issue, unsupervised GAN-based methods have gained attention for their ability to learn without paired data. EnlightenGAN [28] leverages unpaired low-light and normal-light images with carefully designed discriminators and loss functions for effective enhancement; FlexiCurve [29] and NeRCo [30] also employ adversarial learning to guide the generator; CLIP-LIT [31] combines cross-modal supervision to learn prompts from images under different lighting for enhancement. However, unsupervised GAN methods are sensitive to the choice of unpaired training data and can suffer from instability.

To combine the strengths of paired supervision and adversarial learning, Yang et al. [32] proposed a two-stage semi-supervised model: The first stage learns coarse-to-fine frequency-band representations from paired data, and the second stage reorganizes these representations through adversarial learning. While effective in improving generalizability, this approach still faces the risk of overfitting to paired data and has high memory consumption.

To address the limitations of existing methods, we propose an innovative strategy: By leveraging the distinct variation patterns of illumination and reflectance components in low-light images, we achieve their decoupled estimation. Specifically, the illumination component is adaptively estimated using curve-fitting techniques, while the reflectance component is enhanced through targeted image processing. This method accurately reconstructs the illumination and reflectance components under normal lighting conditions, producing visually natural and artifact-free enhancement results. Furthermore, this approach significantly improves brightness, contrast, and detail preservation, making it particularly suitable for real-world scenarios where paired training data are difficult to obtain.

## 3. Method

### 3.1. Framework Overview

**Figure 2:**
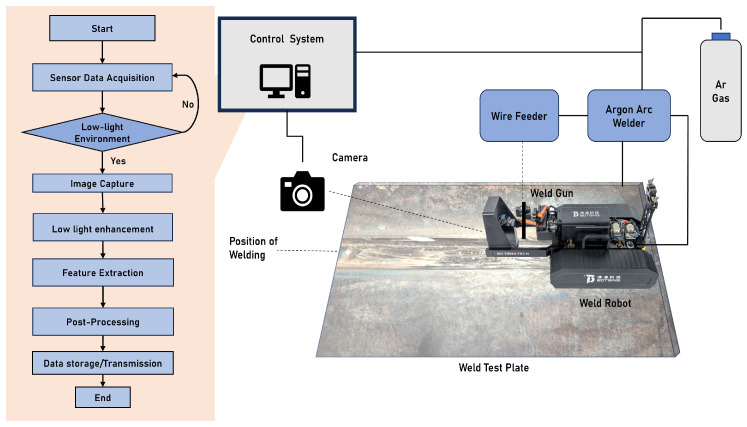
The enhancement process of low-light weld seam images and a schematic diagram of a welding crawler robot: the image enhancement workflow for low-light weld seams, along with a schematic illustration of the welding crawler robot.

Figure 2 illustrates the integration of the proposed low-light weld image enhancement algorithm into the visual control system of a trackless crawling welding robot. The experimental platform, independently developed by Beijing Boxin Technology Co., Ltd. (Beijing 100176, China), comprises three primary components: a dedicated camera, the trackless crawling welding robot, and an industrial control computer.

**Figure 3:**
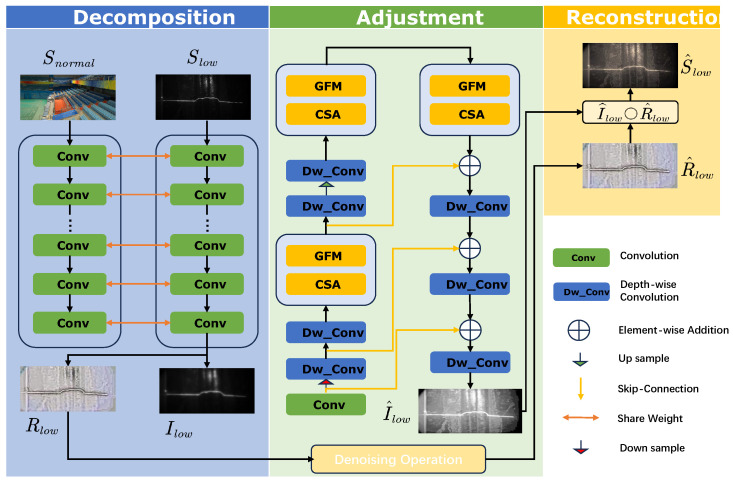
The proposed network enhances low-light images through three stages: It decomposes the input into illumination and reflectance maps, adjusts them via curve estimation and denoising, and then fuses the results to produce the final enhanced image.

The overall architecture of the Retinex-based Illumination Curve Network (RICNET) is depicted in Figure 3. The framework consists of two principal modules. The first is a data-driven decomposition network (DD-Net), grounded in Retinex theory, which decomposes an input image into its illumination and reflectance components. The second is an unsupervised illumination curve estimation network (UICE-Net) that models and adjusts the illumination component through multi-scale feature extraction and attention mechanisms, thereby enhancing illumination correction accuracy. The corresponding algorithmic workflow of RICNET is detailed in Algorithm 1.

Additionally, the reflectance component undergoes denoising via filtering operations, effectively suppressing noise while preserving essential texture details and improving overall visual quality.    
**Algorithm 1:** Low-Light Image Enhancement via Decomposition and Adjustment.     **Input:** Low-light image $S_{\mathrm{low}}$     **Output:** Enhanced image ${\hat{S}}_{\mathrm{low}}$ 1   $[I_{\mathrm{low}},R_{\mathrm{low}}]\leftarrow$
Decomposition Network$(S_{\mathrm{low}});$
// Decompose into illumination and reflectance 2   ${\hat{I}}_{\mathrm{low}}\leftarrow$
Illumination Adjustment Network$(I_{\mathrm{low}});$
// Pixel-wise illumination adjustment in
[0,1]
range 3   ${\hat{R}}_{\mathrm{low}}\leftarrow$
Denoising$(R_{\mathrm{low}});$
// Pixel-wise reflectance denoising 4   ${\hat{S}}_{\mathrm{low}}\leftarrow {\hat{R}}_{\mathrm{low}}\cdot {\hat{I}}_{\mathrm{low}};$
// Reconstruct enhanced image 5   **return  **
${\hat{S}}_{\mathrm{low}}$


The Retinex theory serves as the theoretical foundation of the framework, modeling human color perception by assuming that an observed image *S* can be factorized into reflectance *R* and illumination *I* components:

(1) S=R∘I,

where ∘ denotes element-wise multiplication. Reflectance represents the inherent optical properties of the scene, which are invariant under varying lighting conditions, whereas illumination characterizes the spatially varying lighting effects incident on the scene. In low-light scenarios, degradation in the illumination component manifests as insufficient brightness and uneven spatial light distribution, which the proposed framework aims to rectify.

### 3.2. Data-Driven Decomposition Network

Conventional low-light image decomposition methods rely on hand-crafted constraints to estimate reflectance and illumination maps. Due to the ill-posed nature of Equation (1), these manual constraints often fail to generalize across diverse scenes. To overcome this, we adopt a data-driven approach using deep learning to automatically learn decomposition patterns and improve generalization.

During training, DD-Net takes paired low-light and normal-light images to learn their decompositions, enforcing a shared reflectance component. Although trained on paired data, the network can decompose low-light images independently during inference. It does not require ground-truth reflectance or illumination but instead incorporates reflectance consistency and illumination smoothness priors via loss functions. Unlike intrinsic image decomposition aiming for accurate intrinsic images, our goal is to learn a representation suitable for illumination adjustment by extracting consistent components between low- and normal-light images.

As shown in Figure 3, DD-Net receives paired inputs $S_{\mathrm{low}}$ and $S_{\mathrm{normal}}$ and estimates corresponding reflectance $R_{\mathrm{low}},R_{\mathrm{normal}}$ and illumination $I_{\mathrm{low}},I_{\mathrm{normal}}$ components. The network uses a series of 3×3 convolutional layers with ReLU and sigmoid activations to map inputs into normalized reflectance and illumination maps.

The total loss function *L* combines reconstruction loss $L_{\mathrm{recon}}$, reflectance invariance loss $L_{\mathrm{ir}}$, and illumination smoothness loss $L_{\mathrm{is}}$:

(2) $$L=L_{\mathrm{recon}}+\lambda_{\mathrm{ir}}L_{\mathrm{ir}}+\lambda_{\mathrm{is}}L_{\mathrm{is}},$$

where $\lambda_{\mathrm{ir}}$ and $\lambda_{\mathrm{is}}$ balance the terms.

Reconstruction loss enforces accurate image recovery,

(3) $$L_{\mathrm{recon}}=\sum_{i,j\in \{\mathrm{low},\mathrm{normal}\}}\lambda_{ij}{∥R_{i}\circ I_{j}-S_{j}∥}_{1},$$

reflectance invariance loss promotes consistent reflectance under varying illumination,

(4) $$L_{\mathrm{ir}}={∥R_{\mathrm{low}}-R_{\mathrm{normal}}∥}_{1},$$

and illumination smoothness loss applies a structure-aware total variation regularization,

(5) $$L_{\mathrm{is}}=\sum_{i\in \{\mathrm{low},\mathrm{normal}\}}∥\nabla I_{i}\circ \exp\left(-\lambda_{g}\nabla R_{i}\right)∥,$$

where ∇ denotes spatial gradients and $\lambda_{g}$ controls sensitivity to image structures, reducing smoothness constraints near edges.

### 3.3. Unsupervised Illumination Curve Estimation

We propose a lightweight convolutional neural network named UICE-Net for unsupervised illumination curve estimation. The network takes the illumination component map generated byDD-Net as input and progressively extracts both local and global features through a series of convolutional modules, ultimately producing pixel-wise curve parameter maps for adaptive image enhancement. UICE-Net maintains low computational complexity while enabling fine-grained modeling of illumination variations across different regions, thereby effectively improving the overall perceptual quality of the enhanced images.

The core structure of UICE-Net consists of seven convolutional layers. The first six layers each contain 32 convolutional kernels of size 3×3 with a stride of 1. Except for the first layer, all others adopt depth-wise convolutions to reduce the number of parameters and computational cost. Each of these layers is followed by a ReLU activation to enhance non-linear feature representation. The seventh layer contains 24 convolutional kernels of the same size and stride, followed by a Tanh activation function that constrains the output parameters within the range [−1,1].

**Figure 4:**
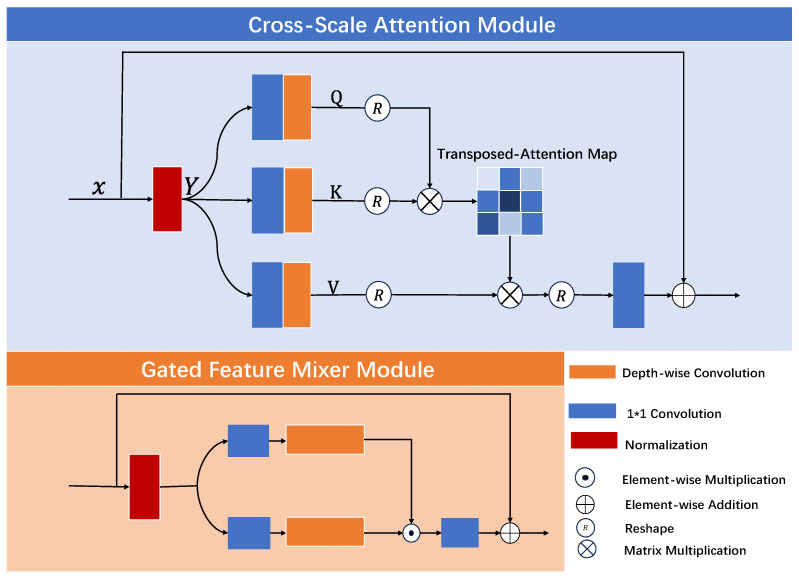
The CSA and GFM modules enhance the network’s ability to handle complex weld seam patterns by fusing multi-scale features and dynamically selecting important information.

To enhance the representation capability of weld seam features, Figure 4 illustrates the integration of the Cross-Scale Attention (CSA) module and the Gated Feature Mixer (GFM) into the convolutional layers. CSA captures contextual information across different receptive fields through multi-scale feature branches and employs an attention mechanism to adaptively fuse features from various scales, thereby improving the network’s ability to model spatial details and global structures—particularly effective for weld seam images with significant scale variations. Meanwhile, GFM dynamically regulates feature channels via a gating mechanism to strengthen key feature representations and suppress redundant information. By utilizing dual-path feature extraction and element-wise gating, GFM achieves effective feature selection and nonlinear interactions, further enhancing the network’s representational power and adaptability to complex scenarios.

The network further employs depth-wise separable convolutions to preserve expressive capacity while reducing complexity. Downsampling is implemented via stride-2 convolutions to expand the receptive field, and upsampling is achieved using bilinear interpolation to restore spatial resolution.

Finally, the network outputs 24 curve parameter maps, corresponding to 8 iterations, with each iteration generating 3 parameter maps for the RGB channels, respectively. These parameters are applied through residual connections to iteratively adjust pixel values in the input image, thereby adaptively enhancing it and significantly improving brightness and detail visibility under low-light conditions.

#### 3.3.1. Illumination Curve Estimation Loss

The illumination enhancement is formulated as a pixel-wise operation, where each pixel intensity I(x) is transformed by a learnable curve:

LE(I(x);α)=I(x)+α·I(x)(1−I(x)),

where I(x) is the normalized pixel intensity at position *x*, and α∈[−1,1] is a learnable parameter controlling the degree of enhancement.

To increase flexibility, this curve is extended to a pixel-wise adaptive form:

LE(x)=I(x)+A(x)·I(x)(1−I(x)),

where A(x) is a spatially adaptive parameter map that adjusts the curve for each pixel.

#### 3.3.2. Unsupervised Loss Functions

To train UICE-Net without paired ground truth, we designed a set of unsupervised loss functions:

- Spatial Consistency Loss ($L_{\mathrm{spa}}$): Preserves the relative differences between neighboring pixels, encouraging structural consistency between the input image *I* and the enhanced image *Y*:
  $$L_{\mathrm{spa}}=\frac{1}{K}\sum_{i=1}^{K}\sum_{j\in \Omega (i)}{\left(\left|Y_{i}-Y_{j}\right|-\left|I_{i}-I_{j}\right|\right)}^{2},$$
  where *K* is the number of local regions and Ω(i) denotes the set of neighbors of region *i*.
- Exposure Control Loss ($L_{\exp}$): Encourages the enhanced image to have a desired average exposure level *E*, set empirically as 0.6:
  $$L_{\exp}=\frac{1}{M}\sum_{k=1}^{M}\left|Y_{k}-E\right|,$$
  where *M* is the number of local image patches.
- Color Constancy Loss ($L_{\mathrm{col}}$): Enforces consistent color distribution across RGB channels in the enhanced image, reducing color distortions:
  $$L_{\mathrm{col}}=\sum_{(p,q)\in \varepsilon }{\left(J_{p}-J_{q}\right)}^{2},\;\varepsilon =\left\{\left(R,G\right),\left(R,B\right),\left(G,B\right)\right\},$$
  where $J_{p}$ and $J_{q}$ denote the mean intensities of channels *p* and *q*, respectively.
- Illumination Smoothness Loss ($L_{\mathrm{tvA}}$): Regularizes the spatial smoothness of the adaptive curve parameter maps *A*, encouraging spatial coherence and reducing noise artifacts:
  $$L_{\mathrm{tvA}}=\frac{1}{N}\sum_{n=1}^{N}\sum_{c\in \{R,G,B\}}{\left(\left|\nabla_{x}A_{c,n}\right|+\left|\nabla_{y}A_{c,n}\right|\right)}^{2},$$
  where $\nabla_{x}$ and $\nabla_{y}$ denote horizontal and vertical gradients, respectively, $A_{c,n}$ is the parameter map for channel *c* at iteration *n*, and N=8 is the number of iterations.

The total loss is defined as a weighted sum:

$$L_{\mathrm{total}}=L_{\mathrm{spa}}+L_{\exp}+w_{\mathrm{col}}L_{\mathrm{col}}+w_{\mathrm{tvA}}L_{\mathrm{tvA}},$$

where $w_{\mathrm{col}}$ and $w_{\mathrm{tvA}}$ balance the contributions of the color constancy and smoothness terms, respectively.

### 3.4. Denoising on Reflectance

In the decomposition step, several constraints are imposed on the network, one of which is the structure-aware smoothness of the illumination map. When the estimated illumination map is sufficiently smooth, all details are retained in the reflectance component, including the amplified noise. Therefore, we can perform denoising on the reflectance before reconstructing the output image using the illumination map. Given that noise in dark regions is amplified according to the illumination intensity, we adopted an illumination-related denoising strategy formulated as

(6) $$\hat{R}=D\left(R_{\mathrm{low}},{\hat{I}}_{\mathrm{low}}\right)$$

where $R_{\mathrm{low}}$ denotes the reflectance component of the low-light image, ${\hat{I}}_{\mathrm{low}}$ is the estimated illumination map of the low-light image, and D(·) is a denoising function that adjusts its strength based on the illumination intensity. The denoising strength is inversely proportional to ${\hat{I}}_{\mathrm{low}}$; that is, the weaker the illumination, the stronger the denoising.

## 4. Experiments

We first introduce the implementation details, evaluation datasets, and performance metrics. Then, we present both quantitative and qualitative comparisons with state-of-the-art methods. Finally, ablation studies are conducted to verify the effectiveness and contribution of each component.

### 4.1. Implementation and Evaluation Details

This section outlines the implementation settings, datasets used for training and evaluation, and the metrics employed to assess model performance.

All models were implemented in PyTorch 1.0.0 and trained on an NVIDIA RTX 3090 GPU (24 GB). The decomposition network (DD-Net) was trained on the LOLv1 dataset [17], which contains 485 training pairs and 15 testing pairs, supplemented by 1000 synthetic pairs to enhance generalization. DD-Net employed a lightweight five-layer convolutional architecture with ReLU activations between most layers to preserve essential features. Training was performed using stochastic gradient descent (SGD) with a batch size of 16 on 96×96 image patches. The loss function consisted of multiple terms weighted by hyperparameters: $\lambda_{\mathrm{ir}}=0.001$, $\lambda_{\mathrm{is}}=0.1$, $\lambda_{g}=10$, and $\lambda_{ij}=0.001$ for i≠j, and $\lambda_{ij}=1$ for i=j. These hyperparameters were empirically determined based on performance on a validation set to effectively balance the contribution of each loss component.

For illumination curve estimation, 2002 images from 360 multi-exposure sequences in SICE Part 1 [33] were used. All images were resized to 512×512 and trained with a batch size of 8. Convolutional weights are initialized with a Gaussian distribution (mean 0, std 0.02), and biases were set to constants. Optimization was performed using ADAM with a learning rate of $1\times 10^{-4}$, and loss weights were $W_{col}=0.5$, $W_{tvA}=20$.

We evaluated our model on several standard benchmarks, including LOLv1 [17], LOLv2 [34] (115 image pairs in total), and a custom weld seam dataset with 112 low-/normal-light pairs. For further validation, we used the Part2 subset of the SICE dataset [33], which contains 229 multi-exposure sequences. Following [33], we selected the first three or four low-light images (depending on sequence length) from each sequence and paired them with the reference image, resizing all images to 1200 × 900 × 3. This resulted in 767 paired low-/normal-light images, referred to as the Part2 testing set. Evaluation metrics included PSNR [35,36] and SSIM [37,38,39] for distortion measurement, and LPIPS [40] (AlexNet backbone [41]) for perceptual quality assessment.

### 4.2. Image Enhancement Performance

We compared our method to a diverse set of state-of-the-art low-light enhancement algorithms, including the unpaired learning-based CLIP-LIT [31], several zero-reference approaches such as ZeroDCE [42], ZeroDCE++ [43], RUAS [44], and SCI [45], and the classical supervised method Retinex-Net [17].

**Figure 5:**
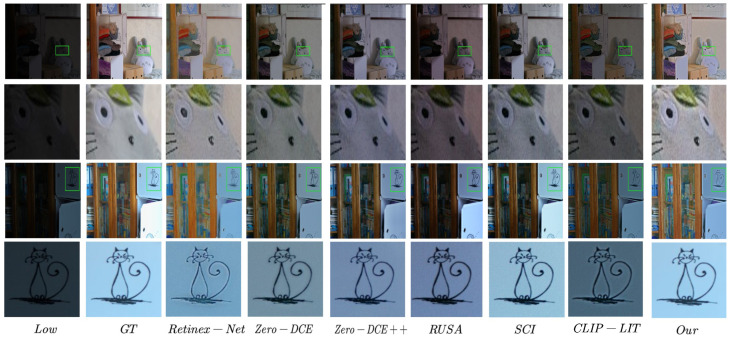
Visual comparisons using the LOL dataset.

As summarized in Table 1 and illustrated in Figure 5, our method was evaluated against this comprehensive range of supervised, zero-reference, and unpaired learning-based enhancement techniques. As summarized in Table 1 and illustrated in Figure 5, we evaluated our method against a variety of supervised, zero-reference, and unpaired enhancement techniques.

The classical supervised method Retinex-Net [17] showed moderate results with PSNR 16.77, SSIM 0.56, and a relatively high LPIPS of 0.47, reflecting challenges in handling complex lighting. Among the zero-reference methods, RUAS [44] achieved strong performance (PSNR 18.23, SSIM 0.72, and LPIPS 0.35), ranking second overall. Other zero-reference methods like SCI [45] and Zero-DCE [42] showed comparable perceptual quality (LPIPS 0.32) but lower fidelity (PSNR and SSIM). Zero-DCE++ [43] exhibited the weakest structural preservation, with SSIM 0.45. The unpaired method CLIP-LIT [31] performed the worst numerically (PSNR 14.82 and SSIM 0.52) but maintained reasonable perceptual quality (LPIPS 0.30). Our method outperformed all others in SSIM (0.79) and LPIPS (0.19), with PSNR (18.88) close to RUAS. This demonstrates superior structural fidelity and perceptual quality, effectively balancing enhancement and texture preservation.

As shown in Table 2, we further compared these methods on the SICE Part2 testing set, which contained 767 low-/normal-light image pairs. Retinex-Net and CLIP-LIT again showed moderate performance. Zero-DCE achieved strong perceptual quality (LPIPS 0.207), while SCI offerred a good balance across metrics. However, our method consistently outperformed all others, achieving the best PSNR (19.97), SSIM (0.812), and lowest LPIPS (0.201), demonstrating its robustness across scenes with diverse exposure levels.

Overall, the zero-reference methods dominated over supervised and unpaired approaches on LOL and SICE, with our method setting a new state of the art in perceptual quality and structural preservation.

**Figure 6:**
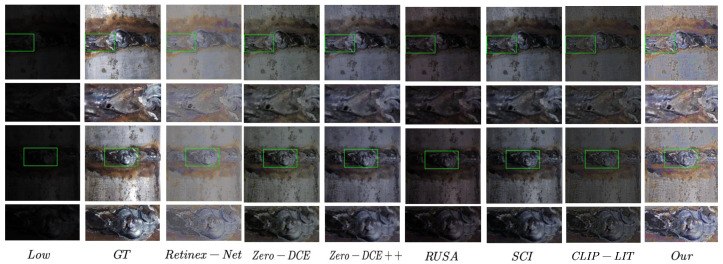
Visual comparisons using the weld dataset.

In the weld seam dataset, as shown in Table 3 and Figure 6, our method also demonstrated superior generalization capability and enhancement quality, making it well-suited for industrial scenarios with challenging illumination conditions. Specifically, we adopted 223 pairs of weld seam images captured under both low-light and normal-light conditions to evaluate the enhancement performance more comprehensively. Here, the low-light condition is defined as environments with ambient illumination below 200 lux, typical of welding and industrial sites where lighting is insufficient and uneven, thus posing significant challenges for image acquisition and feature recognition.

Specifically, our method achieved the highest PSNR (17.38) and SSIM (0.78), along with the lowest LPIPS (0.30), clearly outperforming all baseline methods. These results indicate that our model not only restores image fidelity more accurately but also preserves perceptual quality more effectively, which is critical in complex, low-light industrial environments where accurate feature representation is essential for downstream tasks such as weld seam detection and robot guidance.

Among the baseline methods, Retinex-Net [17], a supervised method, showed relatively strong performance, with PSNR of 16.52 and SSIM of 0.66, but sufferred from a higher LPIPS of 0.40, reflecting limitations in perceptual realism. The zero-reference method Zero-DCE [42] also performed reasonably well (PSNR 12.65, SSIM 0.70, LPIPS 0.38), indicating better generalizability than several other baselines.

However, the other methods struggled significantly. For example, RUAS [44] and Zero-DCE++ [43] exhibited weak structural fidelity and poor perceptual scores, with low SSIM values of 0.37 and 0.35, respectively. CLIP-LIT [31], although based on unpaired learning, also failed to produce competitive results, with PSNR of only 9.19 and LPIPS as high as 0.44. These findings emphasize the challenges of low-light enhancement in real-world industrial data, where many existing methods suffer from insufficient robustness or structural degradation. In contrast, the strong performance of our approach underscores its robustness and adaptability to non-ideal lighting conditions, making it a promising solution for practical deployment in low-light weld inspection and automation tasks.

Table 4 compares different methods in terms of model complexity and runtime. The FLOPs were calculated based on an input image size of 1200 × 900 × 3. Although our method only has 0.15 M parameters, 88.1 G FLOPs, and a runtime of 0.0031 s, demonstrating a good balance between performance and efficiency, Retinex-Net and SCI have similarly small parameter counts but much higher FLOPs—587.47 G and 188.87 G respectively—resulting in significantly lower computational efficiency. While Zero-DCE++ is the most lightweight and fastest method, its enhancement capability is limited. Overall, our method achieved an effective trade-off between complexity and speed, making it more suitable for practical deployment.

### 4.3. Ablation Study

To verify the effectiveness of each component in the proposed UICE-Net architecture, we conducted comprehensive ablation experiments. All models were trained on the publicly available SICE dataset [33] and evaluated on the LOL and weld datasets. Each model was trained for 100 epochs to ensure reliable and fair comparison.

In our ablation setup, we progressively removed individual modules from the full UICE-Net to assess their contribution to the overall performance. The evaluation was performed using the LOL and Weld datasets to demonstrate the model’s generalizability under domain shift. Table 5 presents the results of this study. For placeholder purposes, all metric values were set to zero.

Compared to the baseline, the complete UICE-Net achieved significant performance improvements across all evaluation metrics. PSNR increased from 18.34 to 18.88, which corresponds to a relative improvement of approximately 2.9%. SSIM improved from 0.61 to 0.79, a notable increase of approximately 29.5%, indicating much better preservation of structural information. Meanwhile, the LPIPS score decreased from 0.39 to 0.19, representing a 51.3% reduction, which highlights the enhanced perceptual quality of the output images.

Removing the GFM module led to a drop in SSIM from 0.79 to 0.65 (a 17.7% decrease) and a rise in LPIPS from 0.19 to 0.25 (a 31.6% increase), suggesting that GFM plays a vital role in preserving structure and enhancing visual quality. Similarly, removing the CSA module resulted in PSNR degradation from 18.88 to 18.37 (a 2.7% decrease), and LPIPS worsening from 0.19 to 0.36 (an 89.5% increase), indicating that CSA significantly contributes to both fidelity and perceptual quality. These findings confirm the effectiveness and necessity of each module in the UICE-Net architecture.

In addition to module-wise ablation, we further investigated the impact of the training strategy on model performance. Specifically, we compared two approaches using the SICE dataset: (1) directly training UICE-Net on the raw input images without any preprocessing, and (2) first decomposing images into illumination and reflectance components using Retinex theory, then training the UICE-Net solely on the illumination maps. This comparison aimed to evaluate whether explicit illumination modeling improves low-light enhancement.

All models were trained for 100 epochs under identical conditions. The evaluation on the LOL and weld datasets are summarized in Table 6, which shows that training on Retinex-decomposed illumination maps significantly outperformed direct training on raw images.

These results clearly demonstrate that explicit decomposition of images into illumination and reflectance components via Retinex theory, followed by training UICE-Net on the illumination component, enables the model to more effectively capture and enhance lighting information under low-light conditions. Specifically, PSNR improved from 18.51 to 18.88, representing an increase of approximately 2.0%. SSIM showed a substantial improvement from 0.56 to 0.79, which corresponds to a relative increase of approximately 41.1%. Meanwhile, LPIPS decreased from 0.34 to 0.19, indicating a reduction of approximately 44.1%, reflecting significantly better perceptual quality. These improvements highlight that the decomposition strategy not only enhanced objective image quality metrics but also greatly improved perceptual similarity and structural fidelity. Consequently, this training strategy substantially improved enhancement quality and visual fidelity compared to direct end-to-end training on raw images.

### 4.4. Application to Low-Light Weld Seam Segmentation

To further assess the practical effectiveness of low-light image enhancement, as highlighted in recent reviews and empirical studies [46,47], we evaluated its impact on a downstream task—weld seam segmentation under challenging illumination conditions.Specifically, we constructed a dedicated dataset comprising 5000 training images captured under normal lighting and 683 testing images collected in real-world low-light environments. The segmentation task was performed using the lightweight yet effective PidNet model. To evaluate the segmentation performance, we adopted four widely-used metrics: Intersection over Union (IoU) [48], mean Intersection over Union (mIoU) [49], accuracy [50], and mean accuracy (mAcc) [50], which together provided a comprehensive assessment of pixel-level accuracy and region-level consistency.

**Figure 7:**
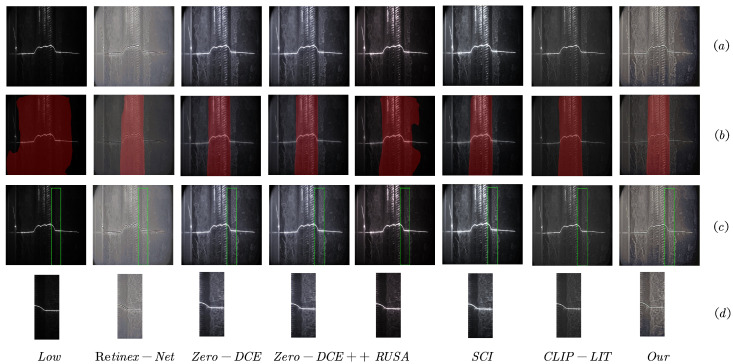
Visual comparisons on weld images captured under low-light conditions. (**a**) Restored images from low-light inputs to normal-light appearance; (**b**) segmentation results based on the restored images; (**c**) selection of local feature regions from the restored images; (**d**) zoomed-in views of the selected local feature regions.

As shown in Table 7 and Figure 7, our method achieved the highest segmentation performance across all metrics. Specifically, we obtained IoU values of 98.20% and 95.10% for the background and weld seam line classes, respectively, with corresponding accuracies of 98.75% and 98.90%. These results significantly surpassed all baseline and state-of-the-art enhancement methods, clearly demonstrating the benefit of our approach in supporting robust downstream vision tasks.

The second-best performer was CLIP-LIT, which also delivered strong segmentation performance but still lagged behind our method. Other methods such as Zero-DCE, Zero-DCE++, RUSA, SCI, and Retinex-Net offerred moderate improvements over the original low-light images but suffered from visual artifacts, insufficient contrast recovery, or structural distortion that negatively impacted segmentation accuracy.

In comparison, segmentation on the original low-light images (i.e., without any enhancement) produced the lowest scores—particularly for the weld seam line class, where accuracy dropped to only 5.97%. This underscores the significant challenge posed by poor illumination in real-world welding scenarios and the necessity of effective enhancement techniques.

To complement the quantitative results, we also present a visual comparison of the enhanced weld seam images captured under low-light conditions in Figure 7. Our method was capable of directly enhancing such images without requiring additional illumination or pre-processing.

As observed in Figure 7, methods like CLIP-LIT and Retinex-Net failed to fully restore edge details, often resulting in blurry or low-contrast outputs. While Zero-DCE, Zero-DCE++, and SCI improved visual clarity and edge contrast, their results often appearred unnaturally bright and deviated from the appearance of weld seams under standard lighting conditions. In contrast, our method produced visually consistent outputs that preserved structural fidelity and maintained natural texture, making it more suitable for industrial applications.

## 5. Discussion

In this paper, we presented the low-light enhancement model RICNET and achieved competitive performance in low-light image enhancement tasks. This paper proposed a zero-reference illumination component estimation network based on the Retinex theory, aiming to achieve effective information restoration of weld seam images under low-light conditions. The focus lies in preserving structural integrity and improving overall visual quality. The proposed network demonstrates strong robustness and generalization capabilities under typical low-light conditions, performing well on both public low-light datasets and real-world welding scenarios, while also maintaining high real-time processing efficiency.

However, under extreme blur or low-contrast conditions, the significant loss of high-frequency details makes structural reconstruction more challenging, thereby limiting the enhancement performance. To improve robustness in complex scenes, future research will explore more efficient zero-reference estimation strategies to enhance adaptability in high-noise and low-contrast environments.

In addition, we plan to integrate adaptive attention mechanisms and hybrid frequency domain modeling on the reflectance map to improve the network’s capability in recovering fine details, especially in capturing and reconstructing high-frequency structural information under extreme conditions. Beyond weld image enhancement, the proposed method also shows good scalability and is expected to be extended to broader industrial vision tasks. Through this research, we aim to promote the transition from task-specific low-light enhancement to a more general image restoration solution, enhancing its practical value and applicability in engineering contexts. 

## Figures and Tables

**Table 1:** Comparison of low-light image enhancement networks to other methods using the LOL dataset.

Type	Method	PSNR ↑	SSIM ↑	LPIPS ↓
Supervised	Retinex-Net [17]	16.77	0.56	0.47
Zero-Reference	Zero-DCE [42]	17.64	0.57	0.32
	Zero-DCE++ [43]	17.03	0.45	0.31
	RUSA [44]	18.23	0.72	0.35
	SCI [45]	16.89	0.54	0.32
Unpaired	CLIP-LIT [31]	14.82	0.52	0.30
Zero-Reference	Our Method	18.88	0.79	0.19

**Table 2:** Comparison of low-light image enhancement networks to other methods using the SICE dataset.

Type	Method	PSNR ↑	SSIM ↑	LPIPS ↓
Supervised	Retinex-Net [17]	19.89	0.783	0.276
Zero-Reference	Zero-DCE [42]	18.69	0.810	0.207
	Zero-DCE++ [43]	16.59	0.720	0.237
	RUSA [44]	13.18	0.734	0.363
	SCI [45]	15.95	0.787	0.235
Unpaired	CLIP-LIT [31]	19.85	0.777	0.289
Zero-Reference	Our Method	19.97	0.812	0.201

**Table 3:** Comparison of low-light image enhancement networks to other methods using the Weld dataset.

Method	PSNR ↑	SSIM ↑	LPIPS ↓
Retinex-Net [17]	16.52	0.66	0.40
Zero-DCE [42]	12.65	0.70	0.38
Zero-DCE++ [43]	11.94	0.35	0.44
RUSA [44]	8.22	0.37	0.39
SCI [45]	11.58	0.65	0.38
CLIP-LIT [31]	9.19	0.53	0.44
Our Method	17.38	0.78	0.30

**Table 4:** Comparison of model complexity and runtime.

Method	Parameters (#)	FLOPs (G)	Runtime (s)
Retinex-Net [17]	0.55 M	587.47	0.12
Zero-DCE [42]	0.08 M	84.99	0.0025
Zero-DCE++ [43]	0.01 M	0.12	0.0012
RUSA [44]	0.03 M	100	0.01
SCI [45]	0.02 M	188.87	0.114
CLIP-LIT [31]	370.01 M	40,568.43	1.95
Our Method	0.15 M	88.10	0.0031

**Table 5:** Ablation study of the proposed UICE-Net using the LOL and Weld datasets. Each variant excluded one key module to evaluate its impact. Gains were computed relative to the baseline.

LOL Dataset						
Variant	PSNR ↑	Gain (%)	SSIM ↑	Gain (%)	LPIPS ↓	Gain (%)
Baseline	18.34	–	0.61	–	0.39	–
w/o CSA	18.37	0.16	0.64	4.92	0.36	7.69
w/o GFM	18.52	0.98	0.65	6.56	0.25	35.90
UICE-Net	18.88	2.94	0.79	29.51	0.19	51.28
**Weld Dataset**						
Variant	PSNR ↑	Gain (%)	SSIM ↑	Gain (%)	LPIPS ↓	Gain (%)
Baseline	16.82	–	0.65	–	0.41	–
w/o CSA	16.90	0.48	0.68	4.62	0.38	7.32
w/o GFM	17.05	1.37	0.70	7.69	0.32	21.95
UICE-Net	17.38	3.33	0.78	20.00	0.30	26.83

**Table 6:** Ablation study comparing training strategies using the LOL and Weld datasets. Each strategy was evaluated with PSNR, SSIM, and LPIPS. Percentage gains were computed relative to the baseline: direct training on raw images.

LOL Dataset						
Training Strategy	PSNR ↑	Gain (%)	SSIM↑	Gain (%)	LPIPS ↓	Gain (%)
Direct training on raw images	18.51	–	0.56	–	0.34	–
Retinex illumination maps	18.88	2.00	0.79	41.07	0.19	44.12
**Weld Dataset**						
Training Strategy	PSNR ↑	Gain (%)	SSIM↑	Gain (%)	LPIPS ↓	Gain (%)
Direct training on raw images	16.85	–	0.70	–	0.35	–
Retinex illumination maps	17.38	3.14	0.78	11.43	0.30	14.29

**Table 7:** Performance comparison of the different enhancement methods on weld seam segmentation.

Enhancement Method	Class	IoU (%)	Accuracy (%)	mIoU (%)	mAcc (%)
Origin	Background	34.41	34.41	35.44	20.19
	Line	36.46	5.97		
Retinex-Net [17]	Background	76.85	80.10	69.30	84.15
	Line	61.75	88.20		
Zero-DCE [42]	Background	85.27	87.06	77.99	90.80
	Line	70.73	94.55		
Zero-DCE++ [43]	Background	82.10	85.50	75.50	88.90
	Line	68.90	92.30		
RUSA [44]	Background	80.45	83.20	72.79	86.98
	Line	65.12	90.75		
SCI [45]	Background	78.30	81.40	70.68	85.50
	Line	63.05	89.60		
CLIP-LIT [31]	Background	97.80	98.46	96.08	98.36
	Line	94.36	98.25		
Our Method	Background	98.20	98.75	96.65	98.83
	Line	95.10	98.90		

## Data Availability

The data presented in this study are available on request from the corresponding author.
